# Management continuity in local health networks

**DOI:** 10.5334/ijic.682

**Published:** 2012-04-13

**Authors:** Mylaine Breton, Jeannie Haggerty, Danièle Roberge, George K Freeman

**Affiliations:** Faculty of Medicine, University of Sherbrooke, Centre de recherché de l’hôpital Charles-Lemoyne, Campus Longueuil, 150 Place Charles-Lemoyne, Bureau 200, Longueuil (Québec), Canada J4L 0A8; Department of Family Medicine, Pine 517, Montreal, Quebec, Canada H2W 1S4; Centre de recherché de l’hôpital Charles-Lemoyne, Campus Longueuil, 150 Place Charles-Lemoyne, Bureau 200, Longueuil (Québec), Canada J4L 0A8; Imperial College London, South Kensington Campus, London SW7 2AZ, UK

**Keywords:** management continuity, patient perspective, coordination of care

## Abstract

**Introduction:**

Patients increasingly receive care from multiple providers in a variety of settings. They expect management continuity that crosses boundaries and bridges gaps in the healthcare system. To our knowledge, little research has been done to assess coordination across organizational and professional boundaries from the patients’ perspective. Our objective was to assess whether greater local health network integration is associated with management continuity as perceived by patients.

**Method:**

We used the data from a research project on the development and validation of a generic and comprehensive continuity measurement instrument that can be applied to a variety of patient conditions and settings. We used the results of a cross-sectional survey conducted in 2009 with 256 patients in two local health networks in Quebec, Canada. We compared four aspects of management continuity between two contrasting network types (highly integrated vs. poorly integrated).

**Results:**

The scores obtained in the highly integrated network are better than those of the poorly integrated network on all dimensions of management continuity (coordinator role, role clarity and coordination between clinics, and information gaps between providers) except for experience of care plan.

**Conclusion:**

Some aspects of care coordination among professionals and organizations are noticed by patients and may be valid indicators to assess care coordination.

## Introduction

Patients increasingly receive care from multiple providers in a variety of settings. As they move from one provider to another, patients expect that services delivered by different providers will be coordinated in a timely and complementary manner such that their care is connected and coherent [1, 2]. Their perception of care coordination refers to management continuity or cross-boundary continuity [3, 4]. Management continuity refers to a consistent and coherent approach to the management of health conditions through the delivery of complementary services in a timely manner [3, 5]. It is about the processes involved in coordinating and personalizing care to deliver high-quality services [6]. In summary, management continuity can be thought of as the ‘seamlessness’ of care and involves crossing boundaries and bridging gaps in care systems that are increasingly complex and specialized [6, 7]. Note that management continuity does not refer to an attribute of healthcare organization, but rather to the perception patients have when they experience services coordination [3]. Since the 1990s, successive reforms have been implemented to improve coordination of services and intensify collaboration between organizations through the creation of integrated health networks in Canada and other countries [8]. Integrated health networks provide coordinated care by ensuring easy links and seamless transitions for patients at various points along the continuum of care [9]. They include a variety of techniques, processes and structures that bring together different providers, formally and informally, to promote the coordination and continuum of care and achieve system efficiencies. The term integrated health network refers to system integration defined by the extent to which the healthcare organization has established and maintains linkages with other parts of the healthcare and social services system to facilitate transfer of care and coordinate concurrent care between different healthcare organizations [2].

According to Freeman and colleagues, experienced continuity is a good outcome of care coordination because it reflects the experience of a coordinated and smooth progression of care from the patient’s point of view [10]. Since patients are the parties experiencing care first-hand from multiple providers (i.e., the patient is the only one present in the different settings), they are uniquely positioned to assess particular aspects of coordination [11]. Measures of management continuity are the least developed of all measures of continuity types, although recently there has been some development [6, 12]. To our knowledge, little research has been done to assess coordination across organizational and professional boundaries from the patients’ perspective [13, 14]. In this paper, our objective is to assess whether greater health network integration, defined by a high degree of inter-organizational arrangements, is associated with better management continuity as perceived by patients. This assessment took place in the context of a major reorganization of the public healthcare system in the Canadian province of Quebec. In 2004, the Quebec government created 95 geographically defined local health networks across the province’s healthcare system, as described below. This reform was intended to enhance coordination of care and to have a positive effect on patients’ experience. In this paper, we look at patients’ perceptions of management continuity, i.e., their experience of care coordination across the local health network from the primary care level.

## Research context

Quebec is a province of over 8 million residents, covered by tax-based system providing universal access to medical services. Institutions such as community health centres and hospitals receive block funding from the Ministry of Health and Social Services, and physicians are reimbursed for services predominantly on a fee-for-services basis.

**Figure 1:**
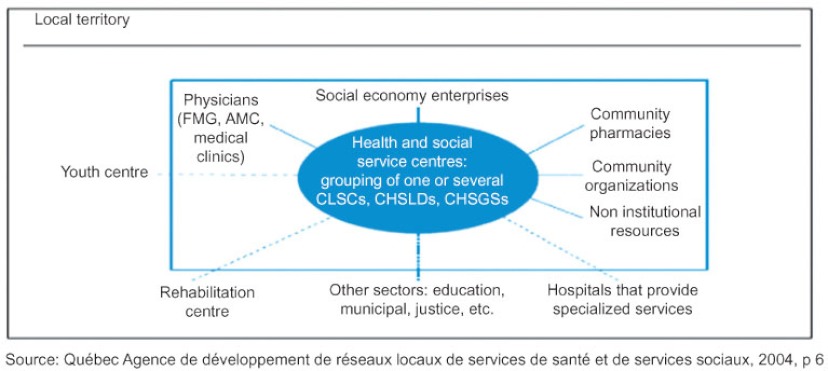
The local health network.

In 2004, the Quebec government initiated a large-scale redesign of its healthcare structure with the aim of implementing local health networks across the province to ensure accessibility, continuity and quality of care. At the heart of the local health networks were 95 new organizations called Health and Social Services Centres (HSSC), shown at the centre of Figure 1, that were created through the merger of several organizations operating within each defined geographical territory: local community services centres (CLSCs, community clinics offering home care and social service), long-term care facilities (CHSLDs) and, in most cases (85%), a community general hospital (CHSGS) [15]. These new HSSCs were formally mandated to lead the implementation of a local health network by encouraging the establishment of formal or informal arrangements between various providers within or outside their territory offering services needed by the local population [16]. The main objective was to increase collaboration with multiple partners such as community organizations, medical clinics and tertiary-care hospitals. HSSCs have a great deal of autonomy in planning and organizing services and activities [17].

## Methods

The data came from a research project on the development of a generic measure of continuity of care, including management continuity. That project’s aim was to develop and validate a generic and comprehensive instrument applicable to a variety of patient conditions and settings. Themes from 33 qualitative studies of patients’ experience with care from various providers were matched to existing instruments to identify potential measures and measurement gaps. Adapted and new items were tested cognitively and the instrument administered to 376 adult patients. In clinic waiting rooms, we found 615 eligible patients, of whom 61% consented to participate, and 79.6% of those (300/376) returned the baseline questionnaire (T1). After initial psychometric analysis, the instrument was modified slightly and administered again to the same patients after a six-month interval. At this time (T2) 85.3% (256/300) responded. These 256 are our sample for this paper. The initial sample size of 300 was estimated for instrument validation as sufficient to obtain stable model resolution of factor analysis with 30–35 items. The response scale for the T2 survey was changed to be able to detect change over time and differences between providers. All subscales of the continuity measurement instrument demonstrated good reliability and measured a single construct [18]. Subscale scores correlated appropriately with indicators of good or problematic continuity.

The self-administered instrument was distributed to adult patients aged 25 to 75 years recruited in the waiting rooms of six primary care clinics. Eligible patients had received care for the same health condition at more than one place in the past year and expected to continue to do so over the next six months. The study population was undifferentiated with respect to health status and had a high likelihood of receiving care at more than one place, although mainly at the primary care level. Patients with serious health conditions such as cancer or severe mental illness were excluded because they principally received specialized services. Since our intention was to create a patient cohort, patients over 75 years old were excluded because they were more likely to be lost to follow-up.

Our generic instrument comprises nine dimensions of continuity [18]: coordinator role*; comprehensive knowledge of patient; confidence and partnership; confidence in team; role clarity and coordination within clinic; role clarity and coordination between clinics*; information gaps between providers*; care plan*; self-information provided.

For this study, we focused on the four dimensions (*) more associated with inter-organizational coordination of care. Table 1 shows these different measures of management continuity, their definition, the subscale construct (α) and the scoring method.

**Figure 2:**
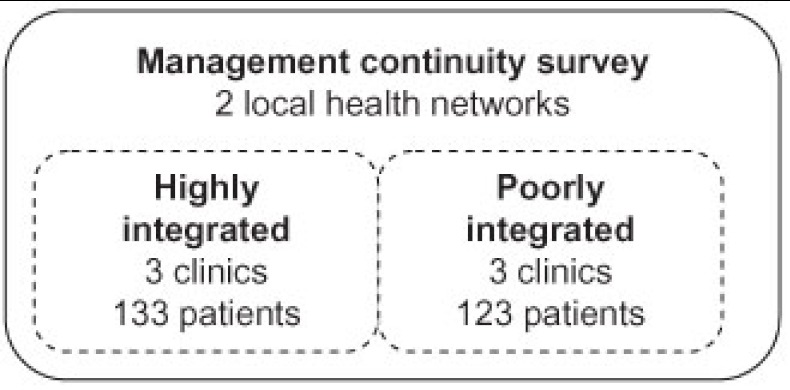
Data source.

To contrast expected continuity, we selected three clinics in highly integrated networks and three in poorly integrated networks (see Figure 2).

Table 2 shows the different characteristics of those two local health networks.

In each network, the clinics selected for our study were typical of the three organizational models operating in the health system. The choice of local health networks was guided by decision-makers’ opinions about the level of integration within the networks.

Telephone interviews were conducted in 23 local health networks (n=23) with executive directors of HSSCs to obtain their perceptions of collaboration between organizations within their local health network, including collaboration between clinics and with the hospital. We chose two networks on the basis of variance and contrast (i.e., low and high collaboration). We then validated this selection using the findings of an organizational survey of 473 primary care organizations in the same 23 local health networks with respect to inter-organizational collaborations [17].

Collaboration was characterized by measuring the formal and informal arrangements between primary healthcare organizations and other health organizations for different activities, as reported by the clinic directors. These arrangements were measured by calculating the percentage of primary healthcare organizations that identified at least one activity with other healthcare organizations within their local health network. As seen in Table 2, the proportion of clinics reporting collaboration between primary healthcare organizations in the highly integrated network was 80% compared with 16% in the poorly integrated network. Also, the proportion of clinics reporting collaboration with the hospital was 93% in the highly integrated network and 22% in the poorly integrated one. The response rate for that organizational survey was 80% (8/10 clinics) in the highly integrated network and 50% in the poorly integrated network (22/44 clinics) [17].

### Analysis

We assessed the correlation between local health network integration and management continuity by using t-tests and χ^2^-testing. Patient characteristics were compared in order to identify potential confounders and interaction effects. All analyses were conducted using SAS 9.2 software.

## Results

The characteristics of the two study populations are shown in Table 3. The patients recruited in the two local health networks were similar. There was no significant difference in age, gender, education, and perception of overall health or financial status—variables known to influence patients’ perception of the quality of care.

### Dimensions of management continuity

The different dimensions of management continuity as perceived by patients in the two local health networks are described in Table 4. For each dimension, we summed the scores of all items and created a local health network mean. The scale and scoring sections in Table 1 present the details of the methodology used for each dimension. For coordinator role and care plan, a higher score indicates higher continuity, and for role clarity and coordination between clinics and information gaps between providers, a higher score indicates lower continuity.

As seen in Table 4, the coordinator role scored significantly higher in the highly integrated network. The role of the coordinator was more extensive and active in the highly integrated network than in the poorly integrated one. Thus, in the highly integrated network, more people reported having a ‘healthcare manager’ who is up-to-date about the care given by other providers and helps them navigate the continuum of care. While the proportion of patients *identifying* a coordinator was somewhat higher in the poorly integrated network (81%, n=100) than in the highly integrated network (74%, n=99), this difference was not significant (p=0.19). Also, patients reported more coordination problems among providers and more information gaps in the poorly integrated network. This suggests the role of the different providers is clearer, with better circulation of information, in the highly integrated network. Finally, for care plan, we found no significant difference between the two networks.

### Experience of management continuity

Ideally, patients should experience excellent management continuity. The best experience of management continuity score indicates the percentage of patients who did not experience any problems of coordination or of information sharing between providers and who reported excellent coordination of care and experience of care plan. We constructed a score for management continuity in the local health networks. The scale and scoring sections in Table 1 specify the cut-off score for each dimension. For example, for high coordination of care we used the proportion of patients who scored 4 or 5 (the maximum). Thresholds for best and worst management continuity experience were based on cut-offs shown in logistic regression models to be sensitive to intermediate outcome variables, such as experience of feeling abandoned, sensing no one was in charge, wanting to change provider, and experience of medical errors. The scales were conceived as ordinal; we dichotomized them to highlight the contrast between the networks.

Table 5 shows the percentage of patients reporting best and worst experiences of management continuity in the two local networks. More patients in the highly integrated network report good management continuity, in all four dimensions, than those in the poorly integrated network.

### Indicators of continuity problems

The continuity measurement instrument also sought to document continuity problems. Table 6 shows the proportion of patients reporting continuity problems. As seen in the table, two indicators were significantly lower in the highly integrated network than in the poorly integrated network; 1) patients feeling no one in the healthcare clinic was really in charge of their care, and 2) patients feeling abandoned by the healthcare clinic.

## Discussion

Numerous advantages have been attributed to the creation of integrated health networks. These include stronger financial performance and greater efficiency, improved quality of care and accountability, improved strategic adaptation to changing conditions, enhanced coordination of care, and improved management of information and clinical standards [19–21]. Besides these effects, our results demonstrated an association between well-integrated health networks and a positive experience of management continuity perceived by patients when encountering multiple care providers in various settings. In our study, eligible patients had received care for the same condition at more than one place in the past year and expected to continue over the next six months, so we could expect management continuity to be an important aspect of their care. We selected patients most likely to need management continuity by virtue of encountering providers in various locations; some patients need more support for coordination than others. Patients most in need of management continuity are likely to be those who, for social or illness reasons, are unable to be their own advocates and who are at risk of experiencing poor quality of care or outcomes as a result of gaps in care.

The results suggest that patients indeed notice the mechanisms of clinical collaboration set up between organizations in local health networks. Two dimensions of management continuity are significantly associated. One is the extent of the coordination role among those with an identified coordinator. We might argue that in a primary healthcare clinic within a well-integrated network, it is easier for the provider to play an active role as healthcare manager for patients, such as helping them get appropriate healthcare from other providers, communicating with other providers as needed, and keeping in touch with patients even when they are receiving healthcare elsewhere. Clinical mechanisms may exist, such as care planning and shared guidelines, to help the provider play an active role in coordinating healthcare. We also found a significant difference in the reporting of information gaps between the health networks, with communication flowing more easily between providers in the highly integrated network. Some communication mechanisms appeared to be more formalized among the members of this network, such as referral of patients to general practitioners, specialists or other professionals and follow-up of the health services they received. Breakdowns in communication mechanisms can affect patients’ confidence in their provider. We found that information gaps and poor coordination significantly predicted whether patients experienced feeling abandoned or that no one was in charge, or would want to change provider. We also found that these factors were related to more serious consequences, namely reports of medical errors and inappropriate use of the emergency room [18].

When we compared patients who reported the best experience of management continuity for all aspects of management continuity, the differences favoured the highly integrated network. This suggests that using stringent evaluation criteria makes the instrument more sensitive to variations. Also, our results clearly demonstrated that more highly integrated healthcare networks were associated with a reduction in continuity problems. Patients in a network with formal arrangements between members seem less likely to feel abandoned by the healthcare system or that no one is in charge of their healthcare.

Several limitations should be noted. First, our results are derived from a study that was not designed to address this research question. Therefore, some information that would have been interesting was not available. For example, to obtain greater clarity on the roles of all providers, it would have been helpful to have more details about the dimension of coordination problems between providers. However, this may be difficult for patients to evaluate, since much care coordination occurs outside their purview and cannot be measured by them. In general, this limitation would tend to underestimate the extent to which network coordination problems were detected; we would expect a more accurate measure to show greater differences between the two networks. Second, our research was set in a specific organizational context, since the creation of local health networks was quite recent. Also the two networks were of different sizes and settings, one urban and the other rural. It may be easier for patients to have a positive experience of management continuity in a smaller network because fewer professionals and organizations have to be coordinated. Finally, the patients participating in the validation of the continuity measurement instrument were clustered in six medical clinics. We did not assess any association with organizational level, and this could have influenced our results. In a 2005 survey of primary care organizations [17], one of the three clinics in the poorly integrated network of our study scored much higher on collaboration compared with that network’s mean. Thus, if we had excluded this clinic, the observed difference between the two networks in our study would have appeared greater. More work is needed to better understand the influence of organizational characteristics at the clinic level on patients’ experience of coordination.

Our study assessed the association between integrated health networks and management continuity as perceived by patients. Previous studies analyzed continuity in specific care contexts such as chronic pain management [12], diabetes [14] and dementia [22]. To our knowledge, little research has been done to date on continuity in the wider primary healthcare context, which by definition includes community-based ambulatory services undifferentiated by health condition or patient profile. Also, instruments on continuity usually focus on relationships between patients and providers.

## Conclusion

Patients are increasingly likely to receive care from multiple providers in a variety of settings. Our results suggest that patients notice some aspects of coordination of care between professionals and organizations and that these may be valid indicators to assess coordination of care. The patients’ perspective has great potential in studying the impact of health policy. The originality of our research lies in the use of a new continuity measure that represents an important addition to toolkits that assess coordination of care from the patients’ perspective, offering both researchers and policy-makers a tool to evaluate and monitor the impact of healthcare reforms on patients’ experience of care.

## Figures and Tables

**Table 1.**
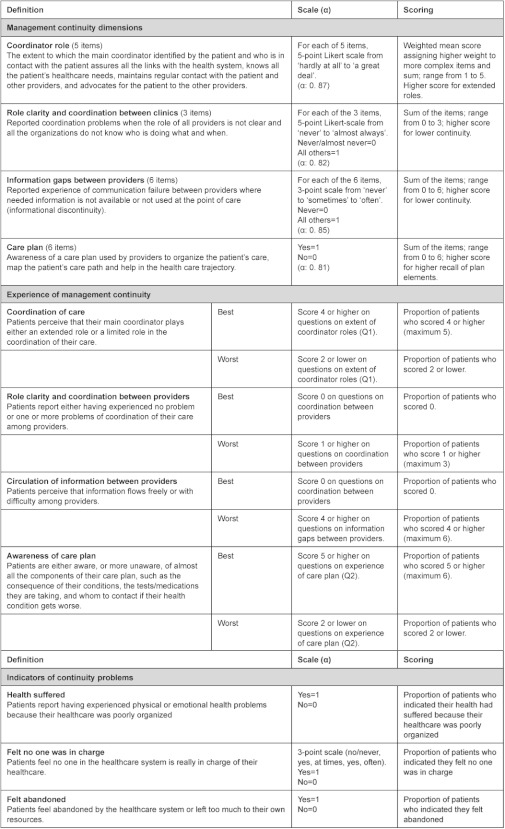
Measures of management continuity

**Table 2.**
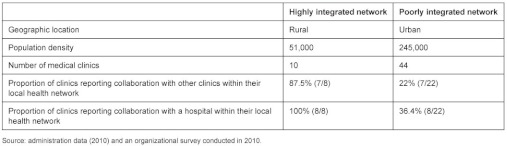
Characteristics of the two local health networks under study

**Table 3.**
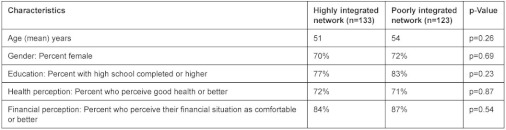
Patient characteristics

**Table 4.**
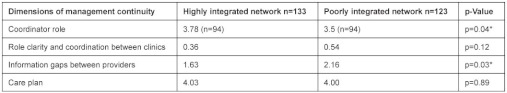
Comparison of highly integrated and poorly integrated networks on dimensions of management continuity

**Table 5.**
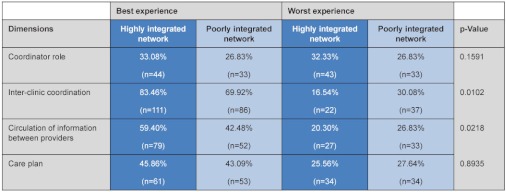
Proportion of patients experiencing the best and worst management continuity by type of network

**Table 6.**
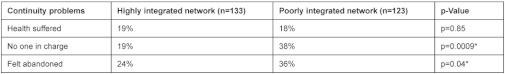
Proportion of patients experiencing continuity problems by type of network
